# Occurrence of triatomines in public spaces: An atypical case in the Southwestern Brazilian Amazon

**DOI:** 10.1590/0037-8682-0042-2023

**Published:** 2023-04-14

**Authors:** Karoline Silva da Cruz, Mariane Albuquerque Lima Ribeiro, Fernanda Portela Madeira, Daniela da Silva Paixão, Adila Costa de Jesus, Luis Marcelo Aranha Camargo, João Aristeu da Rosa, Jader de Oliveira, Paulo Sérgio Bernarde, Dionatas Ulises de Oliveira Meneguetti

**Affiliations:** 1 Universidade Federal do Acre, Programa de Pós-Graduação em Ciências da Saúde na Amazônia Ocidental, Rio Branco, AC, Brasil.; 2 Universidade Federal do Acre, Centro de Ciências da Saúde e do Desporto, Campus Rio Branco, Rio Branco, AC, Brasil.; 3 Universidade de São Paulo, Faculdade de Saúde Pública, Laboratório de Entomologia em Saúde Pública, São Paulo, SP, Brasil.; 4 Universidade Federal do Acre, Centro Multidisciplinar, Campus Floresta, Cruzeiro do Sul, AC, Brasil.; 5 Instituto Nacional de Epidemiologia da Amazônia Ocidental, Porto Velho, RO, Brasil.; 6 Centro de Pesquisa em Medicina Tropical de Rondônia, Porto Velho, RO, Brasil.; 7 Centro Universitário São Lucas, Departamento de Medicina, Porto Velho, RO, Brasil.; 8 Universidade de São Paulo, Instituto de Ciências Biomédicas 5, Monte Negro, RO, Brasil.; 9 Universidade Estadual Paulista “Júlio de Mesquita Filho”, Faculdade de Ciências Farmacêuticas, Departamento de Ciências Biológicas, Araraquara, SP, Brasil.; 10 Universidade Federal do Acre, Programa de Pós-Graduação Stricto Sensu em Ciência, Inovação e Tecnologia para a Amazônia, Rio Branco, AC, Brasil.; 11 Universidade de São Paulo, São Paulo, SP, Brasil.; 12 Universidade Federal do Acre, Colégio de Aplicação, Rio Branco, AC, Brasil.

**Keywords:** Triatominae, Environment changes, Insect vectors

## Abstract

**Background:**

Triatomines infest atypical public spaces in the Western Amazon.

**Methods:**

Frequent visitors to these spaces captured the insects in the state of Acre, Brazil (Rio Branco and Cruzeiro do Sul).

**Results:**

Six insects were found in a penitentiary, a church, a school, a university, a hospital, and a health center. Five of the insects were adults (three positive for *Trypanosoma cruzi*) and one was a nymph.

**Conclusions:**

This is the first report of triatomine occurrence in schools or churches. These data are important for implementing surveillance strategies and alerting individuals about possible changes in Chagas disease transmission dynamics.

Known as vectors of Chagas disease (CD), triatomine bugs (Hemiptera, Triatominae) are insects that feed on blood through all developmental stages^1^. Several factors contribute to the invasion of these invertebrates into the household environment, such as chemical signals emitted by hosts, artificial light, and nocturnal habits^1^.

Anthropogenic changes influence these vectors and reduced the number of shelters and wild food sources for triatomines leading to the intrusion and invasion of these insects into households and peridomestic environments. This invasion presents a greater risk of human infection by *Trypanosoma cruzi* (Chagas, 1909) (Kinetoplastida, Trypanosomatidae*)* and consequently CD^1^ ^,^ ^2^.

Data on the occurrence of triatomines in domestic environments or public spaces in the Amazon are rare, and this information is important for controlling and monitoring the life cycle of these vectors^2^. Thus, the aim of this study was to report triatomine occurrence in “atypical” public spaces in the state of Acre, Western Amazon, Brazil.

Triatomines were collected between September 2011 and August 2019 in the Brazilian municipalities of Rio Branco, the capital of the state of Acre, and Cruzeiro do Sul, the second most populous city in the state, located in the extreme southwest region of the Amazon (Figure 1).

**FIGURE 1:**
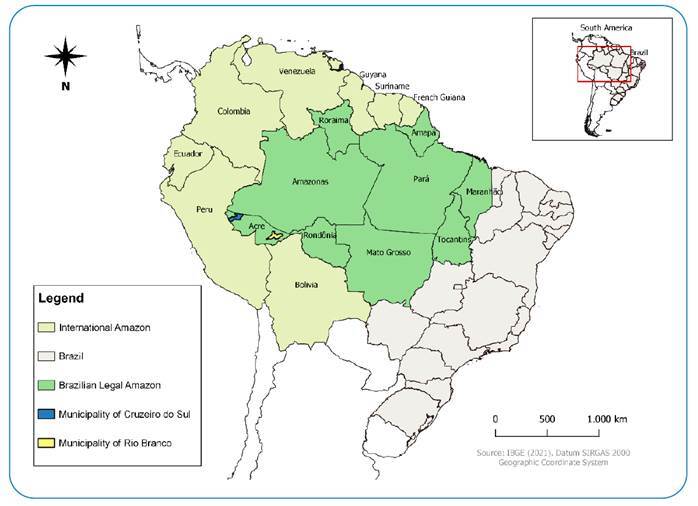
Map of triatomine occurrences in atypical places in the state of Acre.

Triatomines were captured in the following institutional environments in the state of Acre, Brazil: a penitentiary, a health center, a university, a hospital (hemodialysis room), a church, and a school. The capture was carried out by frequent visitors to these public spaces. Insects were delivered to the Entomological Surveillance Divisions of Rio Branco and Cruzeiro do Sul and then sent to the Laboratory of Tropical Medicine (LABMEDT) of the Federal University of Acre (UFAC) in Rio Branco or to the LABMEDT team that operates in the municipality of Cruzeiro do Sul, Acre, Brazil.

Taxonomic identification was based on the key factors described by Lent and Wygodzinsky^3^ and Galvão^1^. Trypanosomatid presence was analyzed in the specimens by investigating the intestinal contents of triatomines based on compression of the abdomen. The material was macerated, diluted in 0.9% saline solution for slide preparation, stained using a quick kit for hematology (0.1% triarylmethane, 0.1% xanthene, and 0.1% thiazine), and then observed under an optical microscope at a magnification of 400x.

Of the insects collected, five were adults and one was a nymph. The identified species were *Rhodnius robustus* (Larrousse, 1927), *Panstrongylus geniculatus* (Latreille, 1811) and *Rhodnius* spp. (*Rhodnius* sp.1 and *Rhodnius* sp.2 - pattern *R. robustus*/*R. montenegrensis* Rosa et al., 2012). *Rhodnius* sp.1 could not be identified at the species level despite the insect being an adult because its genitalia had degraded. The insects identified as *R. robustus* and *P. geniculatus* were positive for trypanosomatids (Table 1 and Figure 2).

**TABLE 1:** Species of triatomines collected and positivity status for trypanosomatids.

Year	Genus/Species	Stage	Trypanosomatid infection	Location	Municipality	n*
2011	*Rhodnius robustus*	Adult	P	Penitentiary	Rio Branco	1
2014	*Rhodnius* sp.1	Adult	N	Health Center	Rio Branco	1
2016	*Panstrongylus geniculatus*	Adult	P	University	Rio Branco	1
2016	*Rhodnius robustus*	Adult	P	Hospital	Rio Branco	1
2018	*Panstrongylus geniculatus*	Adult	N	Church	Cruzeiro do Sul	1
2019	*Rhodnius* sp.2	Nymph	N	School	Rio Branco	1
	(pattern *R. robustus/R.montenegrensis)*					
**Total**						**6**

*n: Sample number.

**FIGURE 2:**
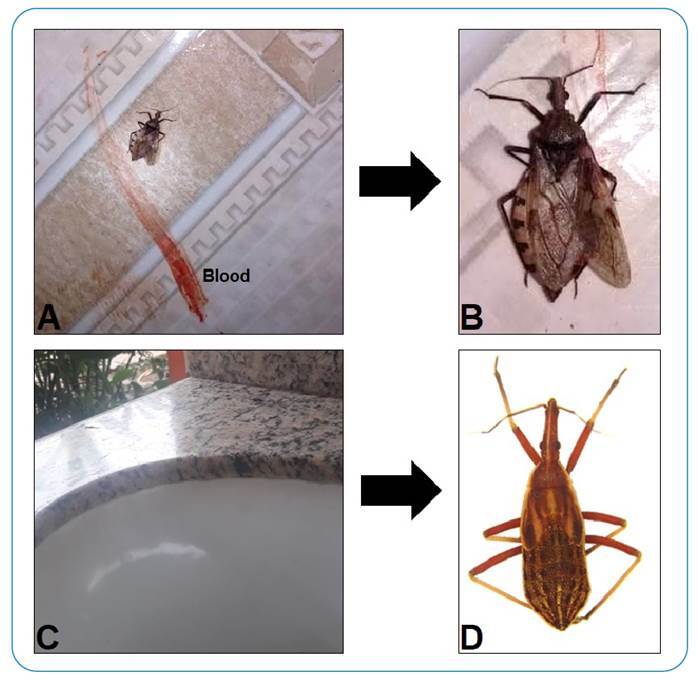
**(A)** and **(B)** A *P. geniculatus* (Latreille, 1811) found dead after a church service in Cruzeiro do Sul, Acre. **(C) and (D)** Nymph of *Rhodnius* sp.2 (*R. robustus*/*R. montenegrensis* pattern) captured in the sink of a children's school cafeteria in Rio Branco, Acre.

In the Western Amazon, one study reported the domiciliation of triatomines in households in the state of Roraima^4^. However, recent studies have reported the intrusion of these insects into urban environments in the states of Acre and Amazonas, Brazil^5^ ^,^ ^6^.

The genus *Rhodnius* Stål, 1859 is among the three main genera demonstrated by epidemiology to transmit CD, and the species belonging to this genus have been frequently associated with the invasion of artificial environments in the Western Amazon^4^ ^-^ ^6^ ^,^ ^12^. Several species were found to be naturally infected by *T. cruzi*, including *R. robustus* ^3^, the first species described in the state of Acre and possibly the first autochthonous case of disease transmission in the state^7^. The species *R. robustus* is found in several specimens of palm trees and epiphytic bromeliads and invades households and performs hematophagy on humans^3^ ^,^ ^8^. Thus, the emergence of such vectors in public spaces can be a risk factor for CD transmission.

Among the species of the genus *Panstrongylus* Berg‎, 1879, *P. geniculatus* is considered a wild vector, is potentially found in birds' nests and in several palm trees, and has been reported in human habitations, possibly because they are attracted to light^3^. In addition, studies have reported insects of the genus *Panstrongylus* in domestic environments and subjects infected by *T. cruzi* in western Amazon^5^ ^,^ ^9^ ^-^ ^12^, and some Latin American countries that border Brazil have reported the colonization of *P. geniculatus* in human dwellings^13^.

Although the presence of *P. geniculatus* in the adult phase has been reported in homes in the state of Acre^5^ ^,^ ^12^, the native natural habitats of this species are still unknown, as all specimens collected so far were at rare encounters. However, the degradation and occupation of forest environments can cause the adaptation of this species to artificial habitats and, subsequently, favor CD transmission^14^.

The triatomine collected in the church was possibly trampled on-site, and it was only identified and handed over to the research team because of the visitor’s prior knowledge of the vector, which highlights the importance of health education as an action to combat CD in the Amazon.

It was not possible to determine whether the insect performed hematophagy on humans or other mammals in the vicinity. However, this species was found to be positive for *T. cruzi* and is responsible for the first autochthonous case report in the state of Rondônia, Brazil, thus raising an alert to the presence of this vector in urban areas^14^.

Reports of adult insects in human dwellings may be increasingly evident due to the destruction of their natural ecotopes^1^ ^-^ ^3^, making monitoring important for detecting changes favoring the domiciliation of some species. In the present study, the spotting of a nymph in an urban, residential area highlighted the importance of this surveillance.

One insect was found on the surface of a sink in a children's school during the day. The school had several palm trees that had been pruned previously, as a form of prevention because an adult triatomine had been found near the school cafeteria. It is important to highlight that the non-identification of a specimen in the nymphal stage is because the reproductive system has not yet fully developed.

The environmental characteristics of the public spaces in this study were similar in terms of the characteristics of the forest fragment, landscaping, and the presence of palm trees in their vicinity, which may have favored the emergence of these insects, mainly of the genus *Rhodnius*, which is directly linked to palm trees^6^ ^,^ ^8^ ^,^ ^13^.

The public spaces where the triatomines were found were urban environments frequented by a large number of people, which is unusual since most insects in published reports were found in residences^3^ ^,^ ^6^ ^,^ ^13^. It is also important to highlight that this study is the first to describe the occurrence of triatomines in schools and churches.

This evidence underscores the importance of reporting the appearance of triatomines in public spaces, to develop surveillance strategies for controlling and monitoring these vectors and to raise awareness of the domiciliation of these insects and possible changes in the dynamics of CD transmission in the region, which has already been observed in other countries^15^.

## ETHICAL CONSIDERATIONS

The collections were conducted under a permanent license issued by the Brazilian Institute of Environment and Renewable Natural Resources (IBAMA). License no. 52260-1.
